# Harmonizing UK primary care prescription records for research: a case study in the UK biobank

**DOI:** 10.1093/jamiaopen/ooag190

**Published:** 2026-10-01

**Authors:** Cai R Ytsma, Ana Torralbo, Natalie K Fitzpatrick, Maik Pietzner, Daniela Nguyen, Ioannis Louloudis, Sabrina Ansarey, Spiros Denaxas

**Affiliations:** Institute of Health Informatics, University College London, London, NW1 2DA, United Kingdom; Institute of Health Informatics, University College London, London, NW1 2DA, United Kingdom; Institute of Health Informatics, University College London, London, NW1 2DA, United Kingdom; Health Data Modelling, Berlin Institute of Health, Berlin, 10117, Germany; Precision Healthcare University Research Institute, Queen Mary University of London, London, E1 1HH, United Kingdom; Precision Healthcare University Research Institute, Queen Mary University of London, London, E1 1HH, United Kingdom; Novo Nordisk Foundation Center for Protein Research, University of Copenhagen, Copenhagen, 2200, Denmark; Institute of Health Informatics, University College London, London, NW1 2DA, United Kingdom; Institute of Health Informatics, University College London, London, NW1 2DA, United Kingdom; Computational Medicine, Interdisciplinary Transformation University (IT: U), Linz, 4040, Austria; British Heart Foundation (BHF) Data Science Centre, Health Data Research United Kingdom, NW1 2BE, United Kingdom; Medical School, Department of Biostatistics, National and Kapodistrian University of Athens, Athens, 10679, Greece

**Keywords:** electronic health records (D057286), epidemiologic studies (D016021), data science (D000077488), prescription drugs (D055553), medical coding (D059019)

## Abstract

**Objective:**

We aimed to develop and validate an automated, scalable framework to harmonize fragmented UK primary care prescription records into a research-ready dataset by mapping diverse medical ontologies to a unified reference standard from the UK Biobank.

**Materials and methods:**

Preprocessing involved selecting a single drug code where multiple were recorded, cleaning codes to match reference presentations, expanding code granularity via drug descriptions, and updating outdated codes. Harmonization mapped British National Formulary (BNF) and Read2 codes to dm+d, the National Health Service standard vocabulary, with records then homogenized to the Virtual Medicinal Product (VMP) granularity. We validated our approach by profiling prescribing patterns across 312 diseases.

**Results:**

We preprocessed 57 659 844 records from 221 868 participants; we dropped 48 950 records for missing drug codes and 13% used multiple ontologies. Most records were BNF-encoded (76%), and 49% required granularity expansion via drug description. In total, 72% of records were harmonized to dm+d, of which 99.98% were converted to VMP. Across 312 diseases, we identified 23 352 disease-drug associations involving 237 medications (BNF subparagraphs) that survived statistical correction, most resembling drug-indication pairs.

**Discussion:**

Our framework addresses a longstanding barrier to large-scale pharmacoepidemiological research by reconciling heterogeneous coding systems into a single, consistent standard. The high conversion rate to VMP demonstrates near-complete coverage, while the recovery of plausible drug-indication associations supports validity. Limitations include residual uncodable records and dependence on dm+d completeness.

**Conclusion:**

Our approach transforms fragmented prescription records into a streamlined, enriched dataset suitable for large-scale discovery research.

## Background and significance

Electronic health records (EHR), data generated as part of clinical care interactions, contain a wealth of information for researchers including detailed medication prescription data. Medication information can help researchers to identify cohorts of people who are prescribed certain drugs, understand trends in prescribing behavior related to shifts in healthcare system policy and reward schemes, or analyze the cost burden of drugs prescribed in primary or secondary care. Prescription data enable scientists to uncover meaningful insights into the health and behavior of the nation, in particular if linked to expensive research data such as whole-genome sequencing.^1^ Additionally, accurate medication prescribing profiles for diseases are a valuable resource that can inform policy makers and accelerate drug discovery by identifying potential drug repurposing opportunities. Medication prescription information however is often fragmented and not stored in a single unified source or format.

Three medical ontologies underpin UK primary care prescription records, each developed for a different purpose. Read version 2 (Read2) is a legacy general-purpose clinical terminology from the 1980s that encodes both diagnoses and prescriptions; it was retired from active NHS use in 2018, but historical records remain widely encoded in it (Table S9). The British National Formulary (BNF) is a hierarchical drug classification designed for prescribing guidance and cost reporting, where the code string itself encodes therapeutic information from broad chapter down to specific presentation. The dictionary of medicines and devices (dm+d), which is the current NHS standard, is built on SNOMED CT and organizes concepts around pharmaceutical relationships between therapeutic moieties, virtual products, and manufacturer-specific actual products. Beyond their differing coding conventions, these ontologies represent drugs at different levels of specificity, map to each other incompletely, and evolve over time with concept identifiers changing across versions. Reconciling them into a research-ready dataset therefore requires principled decisions about ontology priority, granularity alignment, and version reconciliation all of which are the technical basis for the framework we describe here.

In this study, we provide a reproducible methodology for harmonizing primary care prescriptions from England, Scotland, and Wales in UK Biobank (UKB) data by mapping recorded medications to an international standard and a common structure and format. Furthermore, for 312 physical and mental health conditions we provide an open-access resource of contemporary medication prescribing profiles derived from primary care EHR data.

## Materials and methods

### Data sources

We used data from the UKB, a longitudinal study of 500 000 middle aged adults. Volunteers aged 40-69 attended assessment centers in England, Scotland, and Wales between 2006 and 2010 to complete self-reported questionnaire data, undergo baseline assessment of physiological measurements, and consent to sharing of EHR for study follow-up. Of the entire UKB study, primary care prescription records are available for approximately 220 000^2^ individuals with the remaining data for the entire cohort estimated to be released in 2026. The UKB is one of the most widely used research datasets in the world with over 22 000 approved researchers in more than 60 countries.

### Study population

The study population consisted of UKB participants with linkage to primary care data that had at least 1 valid primary care registration period (defined as a valid date of practice registration) in a primary care practice and at least 1 prescription event recorded from July 26, 1945 to August 25, 2019. Most records commenced in the late 1980s; only 279 events recorded between July 1945, 26 and January 1, 1986. Prescription records are recorded in 4 data provider EHR systems.

### Prescription data providers

Data were from 2 primary care data providers in England (The Phoenix Partnership (TPP) and Vision), 1 from Scotland and 1 from Wales. Providers use 1 or 2 out of 3 ontologies to encode their prescriptions (Table 1). UK Biobank receives the data via various suppliers (eg, Albasoft for Scottish data, Secure Anonymized Information Linkage Databank for Welsh data) and performs minimal pre-processing to the raw prescription records.^3^ UK Biobank replaces event dates with dummy dates when (1) the date precedes the participant date of birth (replaced with January 01, 1901), (2) the date matches the participant date of birth (February 02, 1902), (3) the date is within the year of birth of the participant (March 03, 1903), or (4) the date is in the future (July 07, 2037).

**Table 1.** Summary of raw UKB primary care prescription records stratified by ontology and data provider.

Data provider		All records	**dm + d** ^a^	**BNF** ^a^	**Read2** ^a^
**All**	*N* records	57 659 844	6 343 919	43 733 704	14 890 845
	*N* people	221 868	18 271	184 572	51 964
	*N* unique codes	–	13 318	5201	19 522
**1 (England Vision)**	*N* records	6 343 919	6 343 919	–	6 220 687
	*N* people	18 271	18 271	–	18 266
	*N* unique codes	–	13 318	–	10 268
	*N* unique drug names	–	41 794	–	34 094
**2 (Scotland)**	*N* records	4 298 107	–	4 249 157	1 136 887
	*N* people	24 579	–	24 579	13 253
	*N* unique codes	–	–	4566	4514
	*N* unique drug names	–	–	21 415	5580
**3 (England TPP)**	*N* records	39 484 547	–	39 484 547	–
	*N* people	160 018	–	160 018	–
	*N* unique codes	–	–	635	–
	*N* unique drug names	–	–	23 344	
**4 (Wales)**	*N* records	7 533 271	–	–	7 533 271
	*N* people	20 483	–	–	20 483
	*N* unique codes	–	–	–	9579
	*N* unique drug names	–	–	–	–

Abbreviations: BNF, British National Formulary; dm + d, Dictionary of Medicines and devices; TPP, The Phoenix Partnership.

a One entry may record drugs in multiple ontologies. Raw Welsh records using Read2 did not contain any drug names. See Figure S1 for overlap.

### Prescription ontologies

Three ontologies are represented in the UKB primary care prescription data: Read2, dm+d, and BNF. Information about them, including documentation, lookup, and mapping files, is published by the National Health Service (NHS) Business Services Authority (NHSBSA).^4^ A breakdown of ontologies recorded per data provider and their overlap within the raw entries are shown in Table 1 and Figure S1, respectively.

#### Read version 2

Read codes were first developed as a clinical ontology in the early 1980s and were widely used in UK primary care from 1999 to 2018. Read version 2 codes were used for diagnoses and prescriptions until April 2016.^5^ At this time, the NHS migrated to Clinical Terms Version 3 (CTV3) until its last release in April 2018, when SNOMED CT (which includes all CTV3 and Read2 codes by definition) became the official standard for recording information in the NHS.^5^ Read2 codes are formatted as 5 case-sensitive characters: for example, “Paracetamol 500 mg tablets” are encoded as “di21.” ^6^ Read2 codes are used by all UKB data providers except England TPP.

#### SNOMED-CT dictionary of medicines and devices

In 2017, the NHS chose the SNOMED CT-based dm+d as the standard for prescriptions in response to the “lack of standardisation in the UK in describing medicines…and in linking knowledge…to these descriptions.”^7^ Similar to SNOMED CT, concepts in dm+d are represented by a unique numeric SNOMED CT concept identifier.

There are 5 types of dm+d concepts as described in Table S1 and visualized in the NHSBSA data model:^8^ Virtual Therapeutic Moiety (VTM); Virtual Medicinal Product (VMP); Virtual Medicinal Product Pack; Actual Medicinal Product (AMP); and Actual Medicinal Product Pack. In the 2019 UKB primary care data release used here, Vision (England) is the only primary care data provider to encode prescriptions in dm+d, either at the VMP or AMP level. For example, the VMP code for “Paracetamol 500 mg tablets” is 42109611000001109; it has at least 1 associated AMP code, like 17953211000001103 which represents “Paracetamol 500 mg tablets (Phoenix Healthcare Distribution Ltd).”

##### Creating a historically comprehensive dm +_d lookup

Virtual Medicinal Product (and VTM) codes can potentially change over time, for example, when a medication is discontinued or a supplier is renamed. These changes are accounted for at the time of prescribing, but historical records are not retrospectively updated. The NHS Technology Reference Update Distribution (TRUD) regularly releases updated and revised versions of ontologies used by the NHS, including dm+d (SNOMED CT).^4^ To address this challenge, we created a comprehensive dm+d lookup file that included all terminology releases spanning the study period which ended August 25, 2019: 2014 version 1.0.0 (the first dm+d release) to 2024 version 7.2.0 (released at the time of writing).

To develop and streamline a consolidated dm+d lookup file that contains all historical release information we obtained all original dm+d release files from NHS TRUD and deduplicated the lookup concept information while retaining the dm+d version where the concept was first and last mentioned. When dm+d concept identifiers change, records in release files that reference the code are replaced. With this information, we mapped identical drug concepts to their most recent version; a single “reference” code for the concept (Figure S2, Table S2). These reference concepts were also used when mapping BNF and Read2 to dm+d.

#### British national formulary

The BNF is an authoritative reference that provides evidence-based guidance on prescribing, pharmacology, and the safe use of medicines across the UK. British National Formulary is accredited by the National Institute for Health and Care Excellence (NICE) and published by *The British Medical Journal.*^9^ It was used as the basis for the NHS Prescription Services, which calculate and report costs of prescribed and dispensed medications across England,^10^ until the adoption of dm+d in 2019.^11^ British National Formulary code information is available from the NHSBSA Open DataPortal.^12^ Within the UKB primary care prescription data, records from the Scottish and English TPP providers are encoded using BNF codes.

British National Formulary codes are hierarchically organized and expansive, with increasing characters providing increasing detail about the drug. For example, the concept “Paracetamol 500 mg tablets” is coded as 0407010H0AAAMAM: the sequence of characters describes the BNF chapter (04: central nervous system), section (0407: analgesics), paragraph (040701: non-opioid analgesics and compound preparations), chemical substance (0407010H0: Paracetamol), with final characters specifying the presentation of the drug (500 mg tablets).

To map BNF codes to SNOMED CT dm+d codes, we extracted mappings from published mapping data^13^ between BNF codes and all relevant dm+d codes. For our study, we desired a simple 1:1 mapping of BNF to the most appropriate level of information in dm+d. Therefore, we deduplicated the published file by selecting the mapped dm+d code with the most equivalent concept (see Figure S3). Finally, for a small number of remaining unmapped codes we expanded the map file by manually adding equivalent codes as identified by exactly matched descriptions. We then converted the mapped dm+d codes to “reference” dm+d codes as described previously.

### Preprocessing and harmonization

For each prescription event, our framework (Figure 1) implements a 2-step approach consisting of a data preprocessing stage (Figure 1A), and a harmonization stage (Figure 1B). The preprocessing step cleaned raw ontology codes by selecting a single ontology code and aligning it with lookup files where necessary, while the harmonization stage mapped the raw codes to dm+d codes. Descriptions and examples of each of the possible harmonization scenarios are shown in Figure 2. We retained fields with event information such as participant identifier, issue date, and data provider.

**Figure 1.**
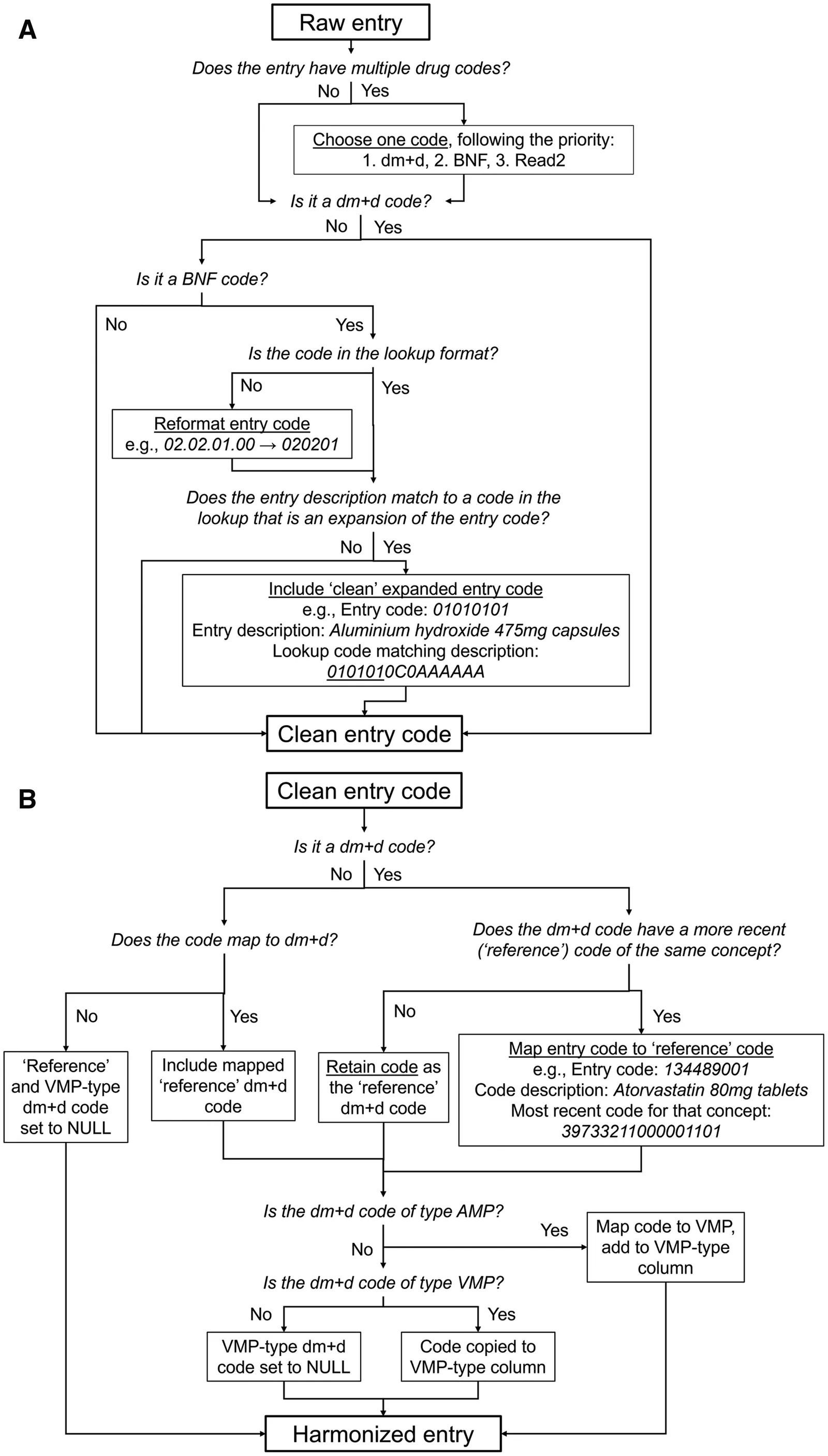
Preprocessing (A) and harmonization (B) method flowcharts for UKB primary care prescription records. By default, prescription codes not found in the lookup files will not have a mapped dm + d code. AMP is the dm + d Actual Medicinal Product, BNF is the British National Formulary, dm + d is the SNOMED CT dictionary of medicines and devices, Read2 is Read version 2, VMP is the dm + d Virtual Medicinal Product.

**Figure 2.**
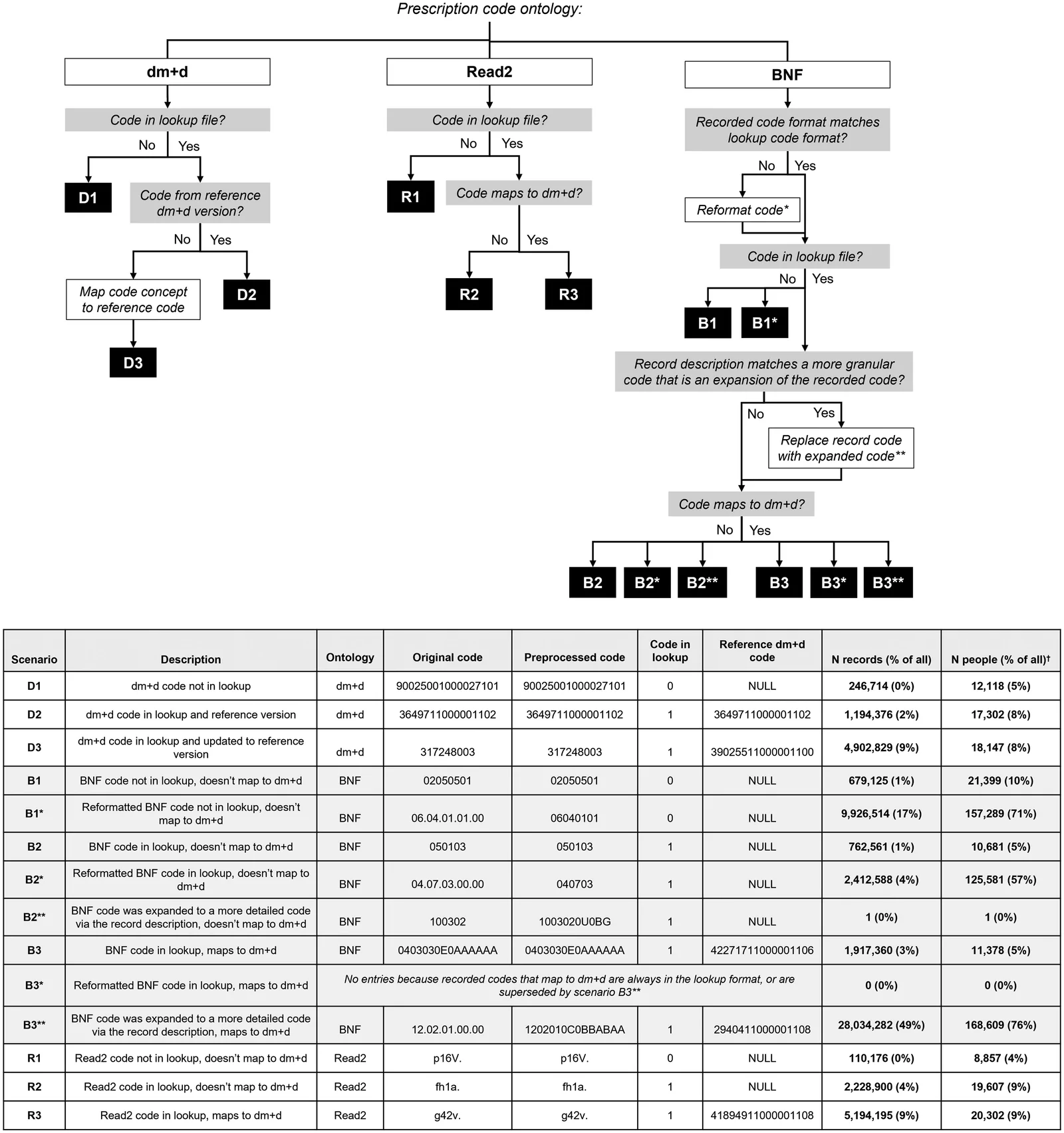
UKB primary care prescription harmonization scenarios per ontology of recorded drug. Reformatting (*) and expansion (**) examples shown in original vs preprocessed codes in the inset table. Expansion supersedes formatting in scenario outcomes. BNF is the British National Formulary, dm + d is the SNOMED CT dictionary of medicines and devices, Read2 is Read version 2.

#### Preprocessing

We utilized the UKB prescription field’s participant ID, data provider, issue date, and drug code(s) from the “gp_prescriptions” table. We used drug name information to expand the granularity of BNF codes in certain scenarios (Figure 1A).

In cases where multiple ontologies were used to record a single prescription event, we implemented a preprocessing step to select the ontology with the highest priority based on a predefined order: (1) dm+d, (2) BNF, and (3) Read2. The priority ontology was selected as dm+d because SNOMED CT is widely used worldwide and contains many well-defined relationships to other SNOMED CT concepts. It is also the NHS standard for medicines and medical devices in England, where the majority of the data originate. While BNF is useful due to its code structure containing innate information about drug components, BNF is limited in interpretability due to UK-only scope. Read2 had the lowest priority because this system is obsolete in the NHS.

Final drug codes were optionally preprocessed further to derive a “clean” entry code, as explained in the following harmonization sections.

#### Read2 harmonization

Read2 codes in English and Scottish entries were not considered because those entries either had BNF or dm+d codes that were preferentially selected to represent the drug concept. We could not locate a single authoritative reference standard for Welsh-based Read2 medication codes as these are locally defined codes that are developed by primary care information system vendors. As a result, we developed a bespoke lookup file for Read2 medication codes to enable their mapping to dm+d. We queried the Unified Medical Language System (UMLS)^14^ and filtered for these codes and their descriptions, as well as any SNOMED CT (dm+d) code that had the same UMLS concept identifier (CUI) as the Read2 code. For Welsh Read2 codes without a UMLS-based dm+d map, we compared the UMLS-based descriptions to reference dm+d descriptions and any exact matches were added to the mapping file after aligning to their reference codes.

#### dm+d harmonization

For dm+d prescription events where the recorded concept was inactive, we forward mapped the concepts to the most recent active concepts as defined in the authoritative lookup (Figure S2; Table S2). The final stage of the process involved converting the mapped dm+d codes to a homogeneous granularity type, VMP. Virtual Medicinal Product codes are the most frequently used dm+d codes used in primary prescriptions and comprise of the medication information, strength, and form. A subset of prescription events is recorded using AMP records which are based on VMP but represent the physical product including manufacturer information. After determining the reference dm+d code for all records, codes already in VMP-type were copied as the “reference VMP code.” AMP-type codes were mapped to their source VMP code using the dm+d lookups. Other types of dm+d codes (VTM; <1% of records) were not mapped and had a NULL value as “reference VMP code.”

#### BNF harmonization

For prescription events recorded in BNF, we firstly reformatted BNF codes to a common format used in the lookup by removing period characters and any zeroes at the end of the code (ie, broader codes where detailed information, like strength or chemical substance, is missing): for example, “06.01.01.00” was reformatted as “060101.” In some occasions, the granularity of the code and the drug described in the description field were at odds. Often the drug description was identical to that of a code in the lookup that was an expansion of the event code. In these cases, we replaced the broad BNF with the more detailed code that matched the description and labeled as the “clean” event code. Codes were not changed if the description was not exactly matched in the lookup, or if it matched to a BNF code that was not an expansion of the original code (ignoring the last character). For example, an entry with a code of “01010101” and a description of “Aluminum hydroxide 475 mg capsules”: the description matches the code “0101010C0AAAAAA,” and because the start of this code is the same as the entry code (ignoring the last character), the broad entry code (with a lookup description of “antacids and simethicone”) is replaced by the more expanded code. Finally, we mapped BNF codes to dm+d where possible.

### Validity assessment through disease-medication profiling

To validate the harmonized dataset and our overall methodology, we created and reviewed medication profiles that displayed contemporary medication prescribing in primary care for 312 physical and mental health diseases, selected to represent the disease burden and healthcare utilization of the English population.^15^^,^^16^ We used previously validated phenotyping algorithms^17^ to identify disease cohorts in UKB using data from self-reported and EHR data sources. For each disease, we matched disease cases to controls by age and sex (Figure 3). For cases and controls, we extracted and analyzed post-diagnosis harmonized prescription records. We removed records for drugs that were prescribed less than 10 times or in fewer than 5 individuals. We compared medication frequency between case and control cohorts by quantifying the number of individuals prescribed each drug (represented as BNF subparagraph) in a 2 × 2 table.

**Figure 3.**
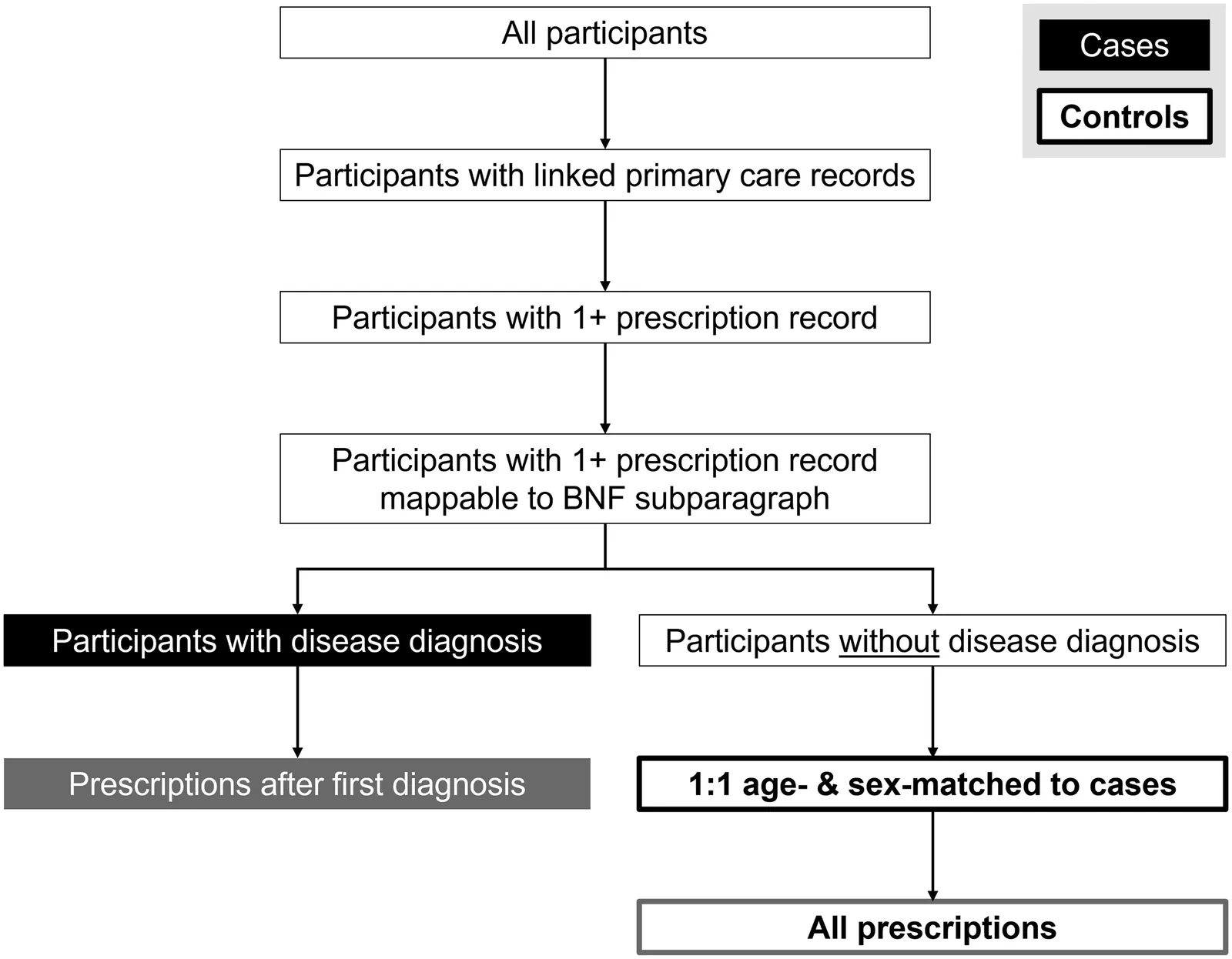
Flowchart of UKB cohort analysis to determine medication profiles following prescription harmonization. Controls contributed all available prescriptions rather than prescriptions after a matched index date as the primary purpose of this analysis is intended as a face-validity check of the harmonization rather than a causal pharmacoepidemiological study. BNF is the British National Formulary.

Controls contributed all available prescriptions rather than prescriptions after a matched index date. This design choice was made because the medication profiles serve as a validation of the harmonization methodology rather than as a causal inference. The absence of an index date for controls means that control prescription histories are, if anything, more comprehensive than those of cases, which would attenuate disease-specific odds ratios (OR) and makes our approach conservative for detecting expected drug-indication signals.

We calculated and reported *P* values and reported OR using 2-sided Fisher’s exact tests. We adjusted *P* values for multiple comparisons using the Benjamini-Hochberg procedure^18^ to control the false discovery rate at 5%. We report the top 10 BNF subparagraphs per disease, the strongest disease-medication associations, and the most commonly co-prescribed medications across all diseases.

### Sample-based accuracy assessment of the BNF expansion procedure

To assess the accuracy of the BNF expansion procedure (Scenario B3**), which accounted for 49% of all harmonized records, we drew a random sample of unique BNF expansion patterns from the framework’s expansion map and retrieved 1 representative prescription record from the harmonized table. One reviewer (S.D.) manually adjudicated each record by comparing the raw drug name (from the source “gp_prescriptions” table) against the expanded BNF description and the resulting reference dm+d code. Records were categorized as (1) fully correct if the raw drug name unambiguously matched the drug represented by the expansion in both identity and dose, (2) correct at the drug and effective-dose level but at a less specific presentation than described in the raw record, (3) a dose error, where the drug and form were correct but the specific strength was differed from that described in the raw record, and (4) incorrect, where a different drug was represented. We calculated and report confidence intervals for the proportion of records in each category using the Wilson score interval.

Data were stored and managed using MySQL (v. 8.4). Analyses were performed using Python (v3.9.25).

## Results

A Sankey diagram (Figure 4) visualizes the overall harmonization procedure and breakdown of different factors/“datasets” indicated A-E: (A) raw records from UKB; (B) preprocessed records; (C) records in B where the drug code is in its ontology lookup file; (D) records in C that have a reference dm+d code; and (E) records in D that map to/are VMP codes.

**Figure 4.**
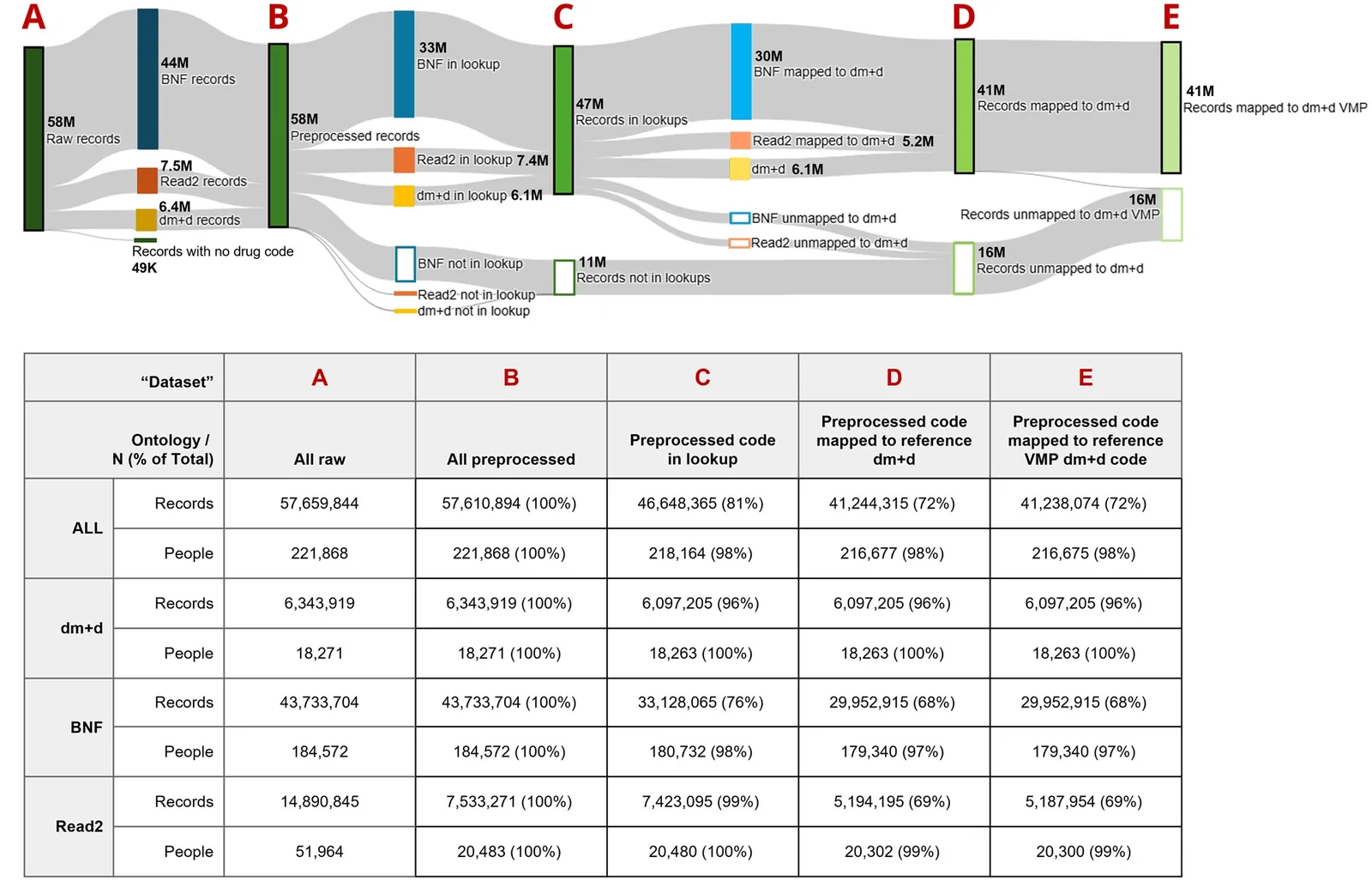
Sankey diagram of harmonization procedure and breakdown of different factors/“datasets” indicated A-E: (A) raw records from UKB; (B) preprocessed records; (C) records in B where the drug code is in its ontology lookup file; (D) records in C that have a reference dm + d code; and (E) records in D that map to/are VMP codes. Row-wise denominators in inset table for datasets B-E are the preprocessed (B) counts. Raw event counts and all people counts are non-exclusive. BNF is the British National Formulary, dm + d is the SNOMED CT dictionary of medicines and devices, Read2 is Read version 2, VMP is the dm + d Virtual Medicinal Product.

### Raw prescription records

From the entire UKB cohort (*N* = 502 131) we identified 221 868 (44%) individuals (55% female) with at least 1 primary care prescription record within a valid primary care registration period. We processed a total of 57 659 844 primary care prescription events: 6 343 919 recorded using dm+d, 43 733 704 recorded using BNF, and 14 890 845 recorded in Read2 codes (Table 1). The highest number of prescription events was contributed from the TPP provider in England (*N* = 39 484 547).

Out of the total 57 659 844 events, the majority of raw prescription records (*N* = 42 596 819; 74%) were coded using BNF alone. We identified 7 357 572 prescription records (13%) that used 2 different ontologies (Figure S1). Specifically, we identified 6 220 687 records (11%) using both Read2 and dm+d and selected the dm+d codes for downstream mapping. Similarly, we identified 1 136 885 records (2%) that contained Read2 and BNF and prioritized BNF codes.

### Prescription harmonization

The application of the harmonization algorithm yielded distinct scenarios across the 3 source ontologies that are detailed below with further examples (Figure 2). The most common harmonization scenario (Scenario B3**) occurred when BNF codes were expanded to a more detailed code via the record description and then mapped to dm+d and was observed in 28 034 282 records (49%). For example, an entry with a BNF code of 12.02.01.00.00 and description of “Beconase Aqueous 50 micrograms/dose nasal spray (GlaxoSmithKline UK Ltd)” was expanded to BNF concept 1202010C0BBABAA, which maps to dm+d code 2940411000001108. Records from England TPP data provider were processed using this scenario as we observed a mismatch in granularity between the total number of unique codes (*n* = 635) and the total number of unique drug names (*n* = 23 344).

### Raw prescription records using dm+d

For events recorded initially using dm+d, 246 714 records (0.004%) were absent from the lookup file (Scenario D1; eg, undocumented code 90025001000027101), while 1 194 376 records (2%) were identified in the lookup and reference version (Scenario D2; eg, 3649711000001102 “Colofac 50 mg/5 ml liquid”). A significant portion, 4 902 829 records (9%), required mapping codes to the reference version (Scenario D3). For example, the concept for “Ranitidine 75 mg/5 ml oral solution sugar free” was represented by 317248003 starting in 2014 (v1.0) but was replaced by 39025511000001100 in 2021 (v3.0) and the latter is therefore used as the reference code.

### Raw prescription records using Read2

For records using Read2, 110 176 (0.002%) could not be found in the lookup (Scenario R1; eg, undocumented code p16V.); however, 2 228 900 records (4%) were present but did not map to dm+d (Scenario R2; eg, fh1a. “Nuvelle TS patch”), while 5 194 195 records (9%) successfully mapped to the target ontology (Scenario R3; eg, g42v. “Clotrimazole 2% cream” maps to 41894911000001108). Of the 20 483 UKB participants with Welsh primary care prescription records, 96.0% (*n* = 19 662) had at least 1 record that did not map to dm+d, while 4.0% (*n* = 821) had complete harmonization of their Welsh prescription records and 0.9% (*n* = 181) had no records that mapped. Across Welsh participants, the proportion of records unmapped to dm+d had a median of 28.3% (IQR, 15.4%-43.8%; 5th-95th percentile, 1.6%-74.3%), meaning that half of Welsh participants retained more than 70% of their prescription record after harmonization. Prescription record volume in this sub cohort had a median of 186 records per participant (IQR, 50-476).

### Raw prescription records using BNF

We observed the highest potential number of harmonization scenarios in BNF due to the formatting and expansion logic. Among BNF codes that did not map to dm+d, 679 125 (1%) failed primarily (Scenario B1; eg, undocumented code 02050501), whereas a substantial 9 926 514 (17%) failed after reformatting (Scenario B1*; eg, 06.04.01.01.00 reformatted to undocumented code 06040101). For BNF codes found in the lookup but not mapping to dm+d, we observed 762 561 records (1%) in the base format (Scenario B2; eg, 050103 “Tetracyclines”), 2 412 588 records (4%) after reformatting (Scenario B2*; eg, 04.07.03.00.00 reformatted to 040703 “Neuropathic pain”), and a single record (1.7E-8%) following expansion (Scenario B2**: 100302 expanded to 1003020U0BG “Voltarol Gel Patch”).

Finally, regarding successful mappings to dm+d from BNF, 1 917 360 records (3%) mapped directly (Scenario B3; eg, 0403030E0AAAAAA “Fluoxetine 20 mg capsules” maps to 42271711000001106) and zero records mapped via reformatting (Scenario B3*), while the expansion logic proved most critical, capturing the largest cohort of the study with 28 034 282 records (49%) successfully mapped (Scenario B3**; as described earlier).

The majority of individuals had at least 1 event code both referenced in the comprehensive lookups and mapped to dm+d (98%); records had 81% and 72% coverage, respectively. Only a small number of records (*N* = 48 950; 0.0008%) were completely dropped from the harmonized table, in cases where there was no drug code reported. Drug descriptions alone were not sufficient for harmonization (Table S3).

Table 2 describes the breakdown of codes and records per data provider following harmonization. A summary of the event and people counts for each of the described datasets is shown in Figure 4. Finally, Table S4 displays the records and people excluded from datasets B-E. People are only included in these counts if all of their prescription records met those specifications. Table S5 shows the breakdown of dm+d types of the reference codes in the dataset D. The last column shows the counts of homogeneous VMP records; effectively all of the information in the reference dm+d dataset can be converted to VMP.

**Table 2.** Breakdown of harmonized records per UKB primary care prescription data provider. dm + d is the SNOMED CT dictionary of medicines and devices.

Data provider	Codes			Records		
	*N* preprocessed	*N* in lookup	*N* reference dm + d codes	*N* preprocessed	*N* in lookup	*N* with a reference dm + d code
**1 (England Vision)**	13 318	10 668	10 668	6 343 919	6 097 205	6 097 205
**2 (Scotland)**	2811	2400	2239	4 249 157	3 508 344	2 690 729
**3 (England TPP)**	6616	6218	6044	39 484 547	29 619 721	27 262 186
**4 (Wales)**	10 002	7727	3302	7 533 271	7 423 095	5 194 195

### Failed harmonization records

Of the 13 780 789 BNF records that failed harmonization to dm+d (24% of all records), 89% were from England TPP (*n* = 12 222 361) and 11% were from Scotland (*n* = 1 558 428), representing 31% and 37% of those providers’ records, respectively. These records failed harmonization either because the encoded BNF drugs were too broad to be mapped to specific medications (eg, 040703 “Neuropathic pain”) for 77% of unharmonized BNF records (*n* = 10 605 639) or because the BNF code was not recognized in the lookup documentation (23%, *n* = 3 175 150). For example, BNF code “02050501” had 1 915 240 entries but is not listed in the BNF lookup file; these entries often had a more specific drug name entered (eg, Lisinopril 5 mg tablets), but because the drugs could not be verified against the unreferenceable and broad BNF code, they were not harmonized. Age and sex distributions of participants with BNF records that failed harmonization to dm+d were consistent with the entire cohort: 45% male, 23% aged 40-49 at baseline, 33% aged 50-59, and 43% aged 60-69. Medication categories (BNF chapters) of these unharmonized records were similar to the distribution in all BNF entries: the cardiovascular system accounted for 33% of all BNF entries vs 28% of unharmonized BNF entries, the central nervous system had 15% vs 5% respectively, gastrointestinal had 9.3% vs 3.4%, endocrine had 11% vs 19%, musculoskeletal and joint diseases had 4.8% vs 3.2%, respiratory system had 6.3% vs 10%, skin had 4.2% vs 6.2%, infections had 3.8% vs 8.4%, and nutrition and blood had 4.0% vs 10%.

As mentioned, the Welsh Read2 records that failed harmonization to dm+d were majority due to a missing map to dm+d (*n* = 2 228 900; 95% of failed Read2 records). Welsh prescription records are comprised of local codes and do not contain drug descriptions, so drug and mapping information was limited to what was identifiable within UMLS. All 20 483 participants with Welsh Read2 prescriptions had both harmonized and unharmonized drug records.

Harmonization failure differed across BNF chapters (Supplementary Figure S5). Within-chapter failure rates ranged from 15% (central nervous system) to 90% (malignant disease and immunosuppression). The latter is expected as the majority of such drugs are administered in secondary care and are not captured in primary care prescription records. Non-medication chapters (which included appliances, stoma appliances, incontinence appliances, and dressings) showed failure rates between 38% and 90%, which is expected. Within medication chapters, failure rates were highest for nutrition and blood (65%), infections (64%), immunological products and vaccines (51%), and endocrine (48%), and lowest for central nervous system (15%), eye (16%), gastrointestinal (17%), and cardiovascular (26%) chapters. Temporal harmonization failure rates varied substantially by data provider (Supplementary Figure S5, top panel), with annual prescription record volumes (bottom panel) providing context to estimate reliability. Across the portion of the study period when all providers contributed >100 000 records per year (∼1995-2013), England Vision failure rates declined from ∼27% to near zero, reflecting Vision’s transition to dm+d as the mandated standard. Wales and England TPP failure rates also declined over this time period (Wales from ∼40% to ∼28%; England TPP from ∼45% to ∼30%), likely reflecting improved coverage of prescription codes in the mapping resources. Scotland was the exception, with failure rates rising from ∼25% to ∼38% across the same period. These patterns imply that analyses restricted to Vision-encoded records or to more recent time windows for Vision benefit from very high harmonization coverage while analyses relying on Scotland records or spanning the full study period across all providers will potentially reflect a temporally patterned unmapped component.

### Sample-based accuracy assessment of the BNF expansion procedure

Of 130 sampled BNF expansion patterns, 122 records (93.8%; 95% Wilson CI, 88.3%-96.8%) were fully correct in both drug identity and dose. Two records (1.5%; 95% Wilson CI, 0.4%-5.4%) represented the correct drug at the correct effective strength but at a less specific presentation level, that is, “Dorzolamide 20 mg/ml eye drops 0.2 ml unit dose preservative free” expanded to “Dorzolamide 2% eye drops,” losing the preservative-free unit-dose presentation while preserving the drug identity and concentration. Two records (1.5%; 95% Wilson CI, 0.4%-5.4%) had a genuine dose error within a graded-dose product family, that is, “Neupro 6 mg/24 hours transdermal patches” expanded to the 4 mg strength of the same product, “Adartrel 500microgram tablets” expanded to the 250 mg strength. Four records (3.1%; 95% Wilson CI, 1.2%-7.6%) represented a different drug from that described in the raw record, that is, “Exjade 125 mg dispersible tablets” expanded to “Eculizumab 300 mg/30 ml solution for infusion vials.” Combining fully correct records with those correct at the drug and effective-dose level, 124 of 130 records (95.4%; 95% Wilson CI, 90.3%-97.8%) correctly represented the raw prescription at the level relevant for cohort definition and therapeutic-class analyses.

### Validity assessment through disease-medication profiling

In 312 physical and mental health conditions, we identified 47 657 associations with 239 unique drugs (represented as BNF subparagraphs). Of those, 23 352 associations (49.00%) survived statistical correction. The median OR of significant associations was 1.10 [IQR, 0.53-2.01] and we observed an OR > 1 in 11 904 associations. Example results for a single disease are displayed for participants with chronic obstructive pulmonary disease (COPD), excluding bronchitis (Figure 5; Table 3).

**Figure 5.**
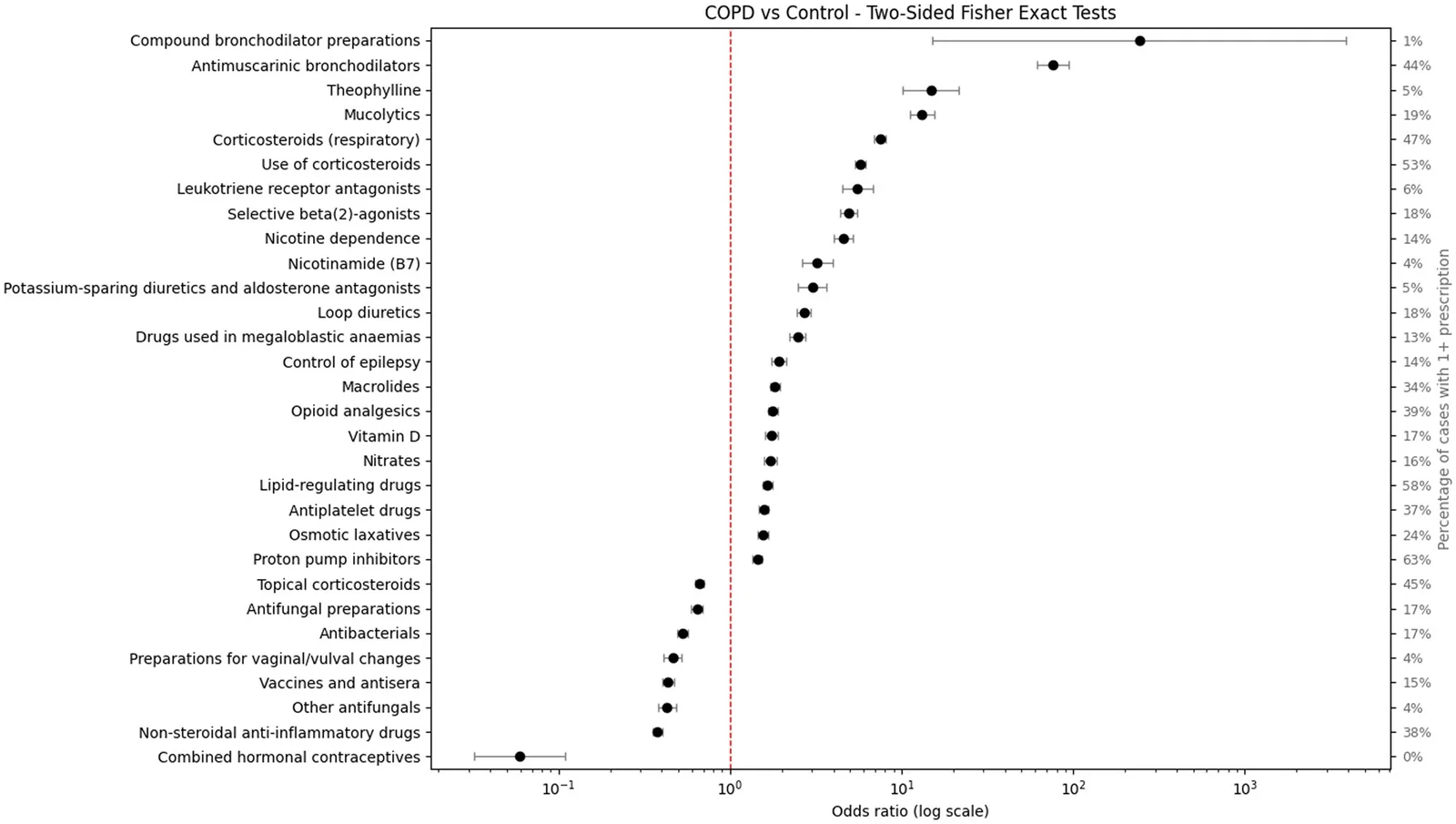
OR of drug categories (via BNF subparagraphs) being prescribed to cases vs controls, via 2-sided Fisher’s exact tests, for example, disease COPD. Two-sided Fisher’s exact tests were performed for 202 BNF subparagraphs, comparing 8956 cases (individuals) against 8956 controls (individuals). These tests analyzed a total of 2 560 769 case records and 1 911 835 control records. Results for 30 associations with lowest *P* values shown.

**Table 3.** Summary of UKB case and age- and sex-matched control cohorts for an example disease, COPD, following the procedure in Figure 3.

	All UKB participants with COPD	COPD with primary care records	COPD with 1+ primary care prescription record AFTER first diagnosis	COPD with 1+ prescription record AFTER first diagnosis mappable to BNF subparagraph (“cases”)
***N* participants**	25 505	12 374	9077	8956
***N* (%) male**	13 872 (54%)	6619 (53%)	4897 (54%)	4837 (54%)
***N* (%) female**	11 633 (46%)	5755 (47%)	4180 (46%)	4119 (46%)
***N* (%) age^a^ <40**	322 (1%)	201 (2%)	195 (2%)	193 (2%)
***N* (%) age^a^ 40-49**	1487 (6%)	865 (7%)	819 (9%)	804 (9%)
***N* (%) age^a^ 50-59**	5569 (22%)	3027 (24%)	2664 (29%)	2627 (29%)
***N* (%) age^a^ 60-69**	10 699 (42%)	5314 (43%)	4207 (46%)	4155 (46%)
***N* (%) age^a^ 70-79**	7371 (29%)	2948 (24%)	1192 (13%)	1177 (13%)
***N* (%) age^a^ 80+**	185 (1%)	66 (1%)	0 (0%)	0 (0%)

Diagnostic code-based algorithm defined at Torralbo et al.^17^

a Age at first diagnosis

The strongest associations between diseases and drugs were observed for Alzheimer’s disease and Drugs for dementia (OR, 6081.7), Dementia and Drugs for Dementia (OR, 3953.8), Parkinson’s disease and Dopaminergic drugs used in Parkinsonism (OR, 1100.4), Type 1 diabetes and Treatment of hypoglycemia (OR, 1049.1), and Myasthenia gravis with Drugs which enhance neuromuscular transmission (OR, 782.6).

The most commonly associated medications shared across diseases were osmotic laxatives (*n* = 231 diseases), vitamin D (*n* = 211 diseases), stimulant laxatives (*n* = 202 diseases), control of epilepsy medication (*n* = 201) and drugs used in megaloblastic anemias (eg, folic acid) (*n* = 199 diseases).

## Discussion

Our methodology reduced fragmentation of dm+d records by converting concepts to their most recent version because VMP codes, the most common type used in primary care (Table S2), can change across different release versions (Table S1).

The large volume of BNF records that were mismatched in granularity between the recorded code and drug description (28 million) also highlight the value of our methodology, which judiciously expands the code granularity to match the described prescription when the broad code encapsulates the more specific named drug. Without this procedure, researchers run the risk of inappropriately matching drugs based on description alone, or omit detailed information if they solely use the broad coded values. This step also enabled mapping to dm+d VMP concepts, which was often impossible at the BNF level encoded in the raw records.

Our harmonization approach relies on deterministic mapping against authoritative reference files combined with rule-based logic for code expansion and selection of the reference term version. Recent approaches have explored machine learning (ML) driven approaches for mapping biomedical terminologies including embedding-based models such as SapBERT.^19^ Such methods could in principle recover a portion of our currently unmapped records. However, we note that embedding-based and generative approaches can produce plausible but incorrect mappings that are difficult to detect at scale, lack the per-record auditability that deterministic mapping against authoritative sources provides, and introduce model- and version-dependent variation that complicates reproducibility across studies and over time. We therefore view ML-assisted mapping as a complementary rather than substitute approach and future workflows could combine both methods.

Medication profiles of diseases reflect contemporary clinical practice. In the top 10 largest associations observed in the data across all diseases (Table S7), we observed the highest enriched associations between Alzheimer’s and drugs for dementia (eg, Galantamine), dementia and dementia medication (eg, donepezil hydrochloride) and Parkinson’s disease and dopaminergic drugs used in parkinsonism (eg, levodopa). Similarly, the analyses identified other cross-indicated therapies such as the use of hormone antagonists in primary prostate malignancies and the prescription of hypoglycemia drugs as adjunctive therapy to insulin to improve glycemic control in individuals with type 1 diabetes. The validation analyses presented here assess the clinical plausibility of the harmonized dataset in aggregate, that is, that expected disease-medication associations are recovered at the cohort level rather than the accuracy of individual code mappings. The recovery of pharmacologically expected associations (eg, dopaminergic drugs in Parkinson’s disease, dementia treatments in Alzheimer’s disease, and the COPD medication profile) provides evidence that the harmonization has not introduced any major systematic errors and constitutes a useful sanity check on the harmonized dataset quality. Formal per-mapping validation would require a manuallyadjudicated gold-standard subset against which the automated mapping could be scored, which we have not undertaken as part of this work. We have, however, attempted to mitigate this gap by reusing authoritative published mapping sources, that is, NHSBSA BNF-to-dm+d mappings, NHS TRUD dm+d release files, and UMLS for Welsh Read2 codes. Similarly, the medications observed for COPD (Figure 5) reflect current clinical practice in UK primary care, eg, highest associations were observed for antimuscarinic bronchodilators (long-acting muscarinic antagonists, such as tiotropium) and theophylline which are the cornerstone of COPD maintenance therapy along with corticosteroids and long- and short-acting beta-2 agonists. The presence of nicotine dependence treatments reflects the management of smoking cessation, and the use of macrolides indicates treatment of acute exacerbations of COPD. Chronic obstructive pulmonary disease has very high cardiovascular comorbidity which is evident by associations with loop diuretics, and vitamin D for heart failure and potassium-sparing diuretics, nitrates, lipid-regulating, and antiplatelet drugs for other CVD conditions. Nonsteroidal anti-inflammatory drugs can worsen bronchoconstrictions and are actively avoided in COPD patients which is illustrated by a negative association.

The most frequently co-occurring medications were those reflecting the systemic consequences of multi-morbidity rather than disease-specific treatments. Laxatives, vitamin D supplementation, and drugs for megaloblastic anemia ranked among the highest, consistent with the high prevalence of polypharmacy-induced constipation, nutritional deficiency secondary to chronic illness and corticosteroid use, and B12/folate depletion driven by metformin, methotrexate, and malabsorption syndromes. Antiepileptic drugs were fifth most commonly associated co-occurring medication reflecting their broad clinical utility across epilepsy, neuropathic pain, bipolar disorder, and anxiety which are all conditions highly prevalent in multi-morbid populations.

Previous studies utilizing the UKB prescription data have primarily been cross-sectional. To our knowledge none have curated a comprehensive drug-wide dataset using longitudinal records reflecting clinical care pathways. Previous work focused on certain disease- or intervention-specific prescriptions and manually filtered the dataset for matching records via string matching of drug names,^20^ curating a list of codes,^21^^,^^22^ or a combination of the 2.^23^ Our work shows that primary care prescribing data can be significantly improved for research purposes by preprocessing and harmonizing drug codes, and how it can then be easily homogenized to a single level of information in the international ontology, dm+d (SNOMED CT). The harmonized dataset is far more comprehensive than common methods for filtering the raw records based on a manually curated drug list, often only of 1 ontology or a list of drug names (example shown in Table S8).

Though this methodology is applied in UK-based ontologies (Read2, BNF) and data, the approach to harmonizing EHR can be applied to other ontologies for mapping to SNOMED CT (eg, expanding published lookup files, simplifying and expanding records based on available information). Converting invalid dm+d codes to a single “reference” version and granularity (VMP) also enables streamlined analyses for any international supplier of dm+d prescription records. The harmonized dataset produced by this work is also more easily translated and directly comparable to other ontologies (eg, Anatomical Therapeutic Code [ATC]) or other nations’ data where SNOMED CT prescriptions are available.

The validation analyses presented here assess the clinical plausibility of the harmonized dataset in aggregate, that is, that expected disease-medication associations are recovered at the cohort level rather than the accuracy of individual code mappings. The recovery of pharmacologically expected associations (eg, dopaminergic drugs in Parkinson’s disease, dementia treatments in Alzheimer’s disease, and the COPD medication profile) provides evidence that the harmonization has not introduced any major systematic errors and constitutes a useful sanity check on the harmonized dataset quality. Formal per-mapping validation would require a manually adjudicated gold-standard subset against which the automated mapping could be scored, which we have not undertaken as part of this work. We have, however, attempted to mitigate this gap by reusing authoritative published mapping sources, that is, NHSBSA BNF-to-dm+d mappings, NHS TRUD dm+d release files, and UMLS for Welsh Read2 codes. The sample-based accuracy assessment of the BNF expansion procedure indicated fully correct expansion in 93.8% of records and correct expansion at the drug and effective-dose level in 95.4%. Residual errors were of 3 types: presentation-level granularity losses without drug or dose error (1.5%), dose errors within graded-dose product families (1.5%), and drug-level errors (3.1%).

The face-validity interpretation of the disease-medication profiles is influenced by several features of real-world prescribing that our analysis was unable to separate. Off-label prescribing, that is, beta-blockers for anxiety introduces disease-drug associations that reflect clinical practice rather than the drug’s ontological classification. Drugs with multiple licensed indications (ie, metformin across type 2 diabetes and PCOS) contribute to disease associations without indication linkage in the underlying raw prescription records. As a result, a single OR reflects the sum of prescriptions across all indications a drug is used for and should be interpreted with this in mind. Additional confounding factors influencing temporal and regional variation in prescribing practices include changes over the study period driven by NICE guideline updates, antibiotic stewardship initiatives, and shifts in specific drug preferences, as well as documented differences in prescribing patterns between England, Scotland, and Wales. While these do not attenuate the strongest associations report (ie, dopaminergic drugs in Parkinson’s disease), they can potentially affect the interpretation of weaker associations and of the cross-disease co-prescribing patterns. The disease-drug profiles should be interpreted as real prescribing behavior rather than clean disease to treatment indication maps. These challenges however can and should form the direction for future research and our framework enables these through the provision of a harmonized dataset.

This study has limitations. Many prescription records (3.1M, 6%) were recorded in the raw data using broad BNF levels that do not allow for harmonization to dm+d concepts or included codes that were unable to be referenced (11M, 19%). Missing information on local Welsh Read2 codes also led to 2.3M (4%) records unable to be harmonized. However, these records are retained in the harmonized dataset to allow for further study. We must note that the source data describe prescribed drugs, not dispensed medications, so findings from this data are not appropriate to use as determinations of what drugs people take. Harmonization to VMP codes removes manufacturer level information from the prescription records and researchers with supply-chain or manufacturer-specific pharmacovigilance interests should return to the reference dm+d code retained in the harmonized dataset rather than using the VMP-homogenized field. Diagnoses, clinical workflows, and prescription practices are likely to have changed over the study period but given that case ascertainment remains one of the primary use cases of UKB we have not performed temporal validation of the harmonization and have opted to process all data covering the study period. The approach to construct medication profiles potentially overestimates associations for commonly prescribed medications that accumulate with longer observation windows; weakly positive or protective associations should be interpreted with this in mind.

## Conclusions

Despite the value of prescription data for research, multiple problems are frequently encountered when using prescription records, including fragmentation (such as when multiple codes or levels of drug concepts are present) and data quality and completeness (such as incomplete reference files and undocumented or outdated codes) (Table S6). Our methodology addresses these problems by defragmenting and harmonizing the available data via prioritizing, expanding, mapping, and updating codes to a single reference version, thereby enabling population-wide analyses with minimal effort.

We intend this work to serve as a practical reference for researchers undertaking primary care prescription harmonization, and we have released the analytical code and mapping resources to support direct reuse. Our framework is well-suited to studies where the analytical unit is the drug product at prescribing-level granularity such as defining cohorts of individuals receiving a particular drug, pharmacoepidemiological discovery, understanding prescribing patterns, and disease-drug association analyses. Our framework and the harmonized data are also an appropriate foundation for downstream conversion to other international standards such as ATC or RxNorm, since dm+d VMP provides a stable reference point. Studies requiring dose or quantity information for pharmacokinetic modeling or adherence estimation or studies requiring manufacturer-specific information will need to return to the raw prescription events, as our framework harmonizes drug identity rather than prescribed quantity. For the broad category of population-scale research questions that motivate most use of UK primary care EHR data, our approach can be utilized as a default starting point over ad-hoc description-based filtering, which we and others have shown to be less comprehensive and less reproducible.

Harmonization results show the increased value of conducting this procedure to both enrich the records supplied by UKB and also convert them to a single format that allows for the utilization of the sophisticated SNOMED CT relationship system. Via the provided references to lookup and mapping files generated by this work, we hope that researchers who work with UKB or other sources of primary care prescriptions can use our method to enable further applications of this resource for public good.

## Supplementary Material

ooag190_Supplementary_Data

## Data Availability

We are unable to directly share source data from the UK Biobank. Researchers can apply directly for access which is granted following protocol approval. To request access to UKB data, researchers need to apply directly to the UK Biobank, register in their access management system and submit a research study protocol https://www.ukbiobank.ac.uk/enable-your-research/apply-for-access. Analytical scripts reported in this manuscript have been made available as open source in the repository: https://github.com/atorralbo/ukbiobank_meds_harmonize. Data from the medication profile analyses is publicly available in the repository: https://github.com/atorralbo/ukbiobank_meds_harmonize.
