# Supplementary material for: Harmonizing UK primary care prescription records for research: a case study in the UK biobank
Source: JAMIA Open. 2026 Oct 1;9(5):ooag190. doi: 10.1093/jamiaopen/ooag190 (PMC13630483; doi:10.1093/jamiaopen/ooag190)
Supplement: Supplementary file 1 [file ooag190_supplementary_data.docx]

# Supplementary Figures

**Figure S1**. Ontologies present in the raw UK Biobank primary care prescription records. In cases where multiple ontologies were present, a single code for the event was chosen following the logic in the inset table. dm+d is the SNOMED CT dictionary of medicines and devices, BNF is the British National Formulary, Read2 is Read version 2.

**Figure S2.** Example of selecting a reference dm+d code to reflect a concept in an entry with an outdated code. dm+d is the SNOMED CT dictionary of medicines and devices.

**Figure S3**. Example of BNF to dm+d code mapping. dm+d is the SNOMED CT dictionary of medicines and devices, BNF is the British National Formulary, VMP is Virtual Medicinal Product, VMPP is Virtual Medicinal Product Pack.

**Figure S5:** Within-chapter harmonisation failure rate for BNF-encoded prescription records. For each BNF chapter, the bar shows the percentage of BNF-encoded records within that chapter that failed to map to a reference dm+d code. Annotations show the failure rate (%) and absolute counts as failed records / total records within the chapter. Chapters are ordered by descending failure rate. Bars are shaded by chapter type: medication chapters (black) and appliance/dressing chapters (grey); the latter contain non-medicinal items (bandages, catheters, ostomy supplies, dressings) that are outside the intended scope of the harmonisation framework and are shown for completeness.

# Supplementary Tables

**Table S1.** Examples of dm+d prescription code types and their occurrence in the raw UKB data. The code types are further described in the dm+d data model (NHS Business Services Authority, 2024).

| **dm+d code type** | **Abbreviation** | **Example code description** | **N UKB records** | **N UKB codes** |
| --- | --- | --- | --- | --- |
| Virtual Therapeutic Moiety | VTM | Amoxicillin | 0 | 0 |
| Virtual Medicinal Product | VMP | Amoxicillin 500mg capsules | 5,070,319 | 3,694 |
| Virtual Medicinal Product Pack | VMPP | Amoxicillin 500mg capsules 21 capsule | 0 | 0 |
| Actual Medicinal Product | AMP | Amoxicillin 500mg capsules (GlaxoSmithKline) | 1,026,886 | 6,974 |
| Actual Medicinal Product Pack | AMPP | Amoxicillin 500mg capsules (GlaxoSmithKline) 21 capsule | 0 | 0 |

**Table S2.** Example of logic for selecting ‘reference’ dm+d codes using the authoritative dm+d lookup. The most recent code for the concept is used as the reference.

| **dm+d code** | **Description** | **Valid years (versions)** | **Previous dm+d code** | **Reference dm+d code** |
| --- | --- | --- | --- | --- |
| 19533611000001102 | Telaprevir 375mg tablets | 2014 (1.0) - 2015 (4.0) | N/A | 41794911000001105 |
| 704467005 | Telaprevir 375mg tablets | 2015 (4.1) - 2023 (5.2) | 19533611000001102 | 41794911000001105 |
| 41794911000001105 | Telaprevir 375mg tablets | 2023 (10.0) - present (2024, 7.2) | 704467005 | 41794911000001105 |

**Table S3.** Examples of raw UK Biobank primary care prescription entries that were not harmonised due to lack of drug code.

| **read_2** | **bnf_code** | **dmd_code** | **drug_name** |
| --- | --- | --- | --- |
| NULL | NULL | NULL | Triptorelin Acetate  Powder and solvent for injection  4.2 mg vial (3 mg Triptorelin) |
| NULL | NULL | NULL | Liquid Paraffin And White Soft Paraffin  Ointment  50 % + 50 % |
| NULL | NULL | NULL | Elasticated Tubular  Bandage Bp  10 cm size F |

**Table S4.** Summary of UK Biobank primary care prescription records excluded following harmonisation. Percentage denominator is ‘all’ counts per labelled dataset (see Figure 4). dm+d is the SNOMED CT dictionary of medicines and devices, VMP is the Virtual Medicinal Product form of dm+d codes.

| **“Dataset”** | **B** | **C** | **D** | **E** |
| --- | --- | --- | --- | --- |
| **N (% of total dataset)** | **Record has no drug code** | **Code not in lookup** | **Code unmapped to dm+d** | **Code unmapped to dm+d VMP** |
| Records | 48,970 (0%) | 10,962,529 (19%) | 16,366,579 (28%) | 16,372,820 (28%) |
| People | 0 (0%) | 3,704 (2%) | 5,191 (2%) | 5,193 (2%) |

**Table S5.** Summary of reference dm+d codes in harmonised UK Biobank primary care prescription records by dm+d type. *Denominator is all records with a reference dm+d code (dataset D in Figure 4). VTM is Virtual Therapeutic Moiety, VMP is Virtual Medicinal Product, and AMP is Actual Medicinal Product.

|  | **VTM** | **VMP** | **AMP** |
| --- | --- | --- | --- |
| N People (% of all*) | 1,127 (1%) | 214,138 (97%) | 181,178 (82%) |
| N Records (% of all*) | 6,241 (0%) | 36,324,022 (88%) | 4,914,052 (12%) |
| N Codes (%) | 76 (0.5%) | 4,620 (35%) | 8,484 (64%) |

**Table S6.** Problems and this study’s solutions to using primary care prescriptions for research. dm+d is the SNOMED CT dictionary of medicines and devices, BNF is the British National Formulary, Read2 is Read version 2.

| **Problem** | **Example** | **Solution** | **Example** |
| --- | --- | --- | --- |
| **Fragmentation** | Multiple drug codes from different ontologies within a single event | Preprocessing | Single prioritised code per event (1. dm+d, 2. BNF, 3. Read2) |
|  |  | Harmonisation | Preprocessed codes mapped to the ‘reference’ dm+d version where possible |
|  | Equivalent concepts encoded at different levels | Homogeneous dataset | All possible records mapped to dm+d concepts at the Virtual Medicinal Product level of drug information. |
| **Data quality and completeness** | Missing or incomplete reference files | This study | Novel, open-source lookup files generated by this process:   - consolidated dm+d lookup & map to single version (‘reference’) of concept codes - expanded BNF to dm+d map - Welsh Read2 lookup file - Welsh Read2 to dm+d map |
|  | Recorded codes cannot be referenced or contain conflicting information | Preprocessing | BNF codes consistently formatted to match lookup |
|  |  |  | Expanded BNF code granularity where event code is abbreviated form of the lookup code matching the event description |
|  |  |  | Simple flag for whether the event code is referenceable in its ontology lookup file |
|  | Outdated codes | Harmonisation | dm+d codes mapped to same concept coded in a single ‘reference’ version |

**Table S7.** Ten highest disease-drug associations observed in UK Biobank primary care prescriptions of participants in various disease cohorts.

| **Disease** | **BNF Subparagraph** | **Drug name** | **Odds ratio (OR)** | **P-value (adjusted)** |
| --- | --- | --- | --- | --- |
| Alzheimer’s | 411000 | Drugs for dementia | 6081.8 | 7.20E-289 |
| Dementia | 411000 | Drugs for dementia | 3953.8 | 0.00E-02 |
| Parkinson’s | 409010 | Dopaminergic drugs used in parkinsonism | 1100.4 | 0 |
| Diabetes Type I | 601040 | Treatment of hypoglycaemia | 1049.9 | 1.23E-132 |
| Myasthenia | 1002010 | Drugs which enhance neuromuscular transmission | 782.7 | 1.32E-48 |
| Autoimmune liver disease | 109010 | Drugs affecting biliary composition and flow | 778.0 | 2.94E-71 |
| Pancreatic cancer | 109040 | Pancreatin | 745.6 | 3.82E-62 |
| Diabetes Type II | 601021 | Sulfonylureas | 732.1 | 0 |
| Diabetes (any) | 601011 | Short-acting insulins | 724.4 | 9.06E-106 |
| Schizophrenia | 409020 | Antimuscarinic drugs used in parkinsonism | 689.6 | 1.31E-83 |

**Table S8.** Contents of raw UK Biobank primary care prescription records ("gp_prescriptions”) matched to ‘Paracetamol 500mg tablets’ via the harmonised table.

| **Field** | **N unique values** | **Examples** |
| --- | --- | --- |
| read_2 | 2 | di21.00; di21. |
| bnf_code | 2 | 04.07.01.02.00; 04070100 |
| dmd_code | 13 | 322236009; 171811000001104; 13429711000001102; 67111000001103; … |
| drug_name | 38 | PARACETAMOL tabs 500mg; Paracetamol 500mg caplets (A A H Pharmaceuticals Ltd); Panadol Advance 500mg tablets (GlaxoSmithKline Consumer Healthcare); … |

**Supplementary Table S9**: Ontologies used to encode prescription records by data provider and time period in UK Biobank primary care data.

| **UK Biobank primary care data provider** | **Country** | **Ontologies used to record drug prescriptions** | **Period of ontology use in UK Biobank primary care data** |
| --- | --- | --- | --- |
| Vision | England | dm+d  Read 2 |  |
| TPP | England | BNF | Entire study period |
| EMIS/Vision | Scotland | Read 2  BNF | Entire study period |
| EMIS/Vision | Wales | Read 2 | Entire study period |

**Supplementary Table S10**: Key NHS ontology transition points during the study period

| **Year** | **Event** | **Relevance to UK Biobank primary care EHR data** |
| --- | --- | --- |
| 2014 | First dm+d release | Earliest version in the comprehensive dm+d lookup (v1.0.0) constructed for this study. dm+d concept identifiers can change across releases; codes recorded from this point onward may reference concepts later superseded in subsequent versions. Vision (England) is the only UKB data provider encoding prescriptions in dm+d. |
| 2016 | Final release of Read V2 | Read2 was used for prescriptions until April 2016. Welsh records are encoded entirely in Read2 and Scottish records use Read2 and BNF. After this date, the Read2 vocabulary was frozen and no new concepts were created. |
| 2018 | Final release of CTV3 | CTV3 used for diagnoses in England TPP. After this date, the CTV3 vocabulary was frozen and no new concepts were created. |
| 2018 | SNOMED CT becomes standard for recording clinical information in primary care | Mandated the use of SNOMED CT (of which dm+d is the medicines component) across English primary care. |
| 2019 | dm+d fully adopted as NHS standard for medicines | Future UKB data releases (estimated 2026 for the full cohort) should contain a higher proportion of dm+d-encoded records. |
| 2020 | SNOMED CT becomes standard for recording clinical information in acute care | Relevant to future linkage studies combining primary and secondary care prescriptions, as both settings would share a common SNOMED CT backbone. |
| 2020 | Read V2 deprecated | Historical records remain encoded in Read2 and are retained in the harmonised dataset. No new Read2-coded records should appear in future UKB data releases. |
| 2020 | CTV3 deprecated | Historical records remain encoded in CTV3. No new CTV3-encoded records should appear in future UKB data releases. |
